# Mechanically Flexible and High-Performance CMOS Logic Circuits

**DOI:** 10.1038/srep15099

**Published:** 2015-10-13

**Authors:** Wataru Honda, Takayuki Arie, Seiji Akita, Kuniharu Takei

**Affiliations:** 1 Department of Physics and Electronics, Osaka Prefecture University, Sakai, Osaka 599-8531, Japan

## Abstract

Low-power flexible logic circuits are key components required by the next generation of flexible electronic devices. For stable device operation, such components require a high degree of mechanical flexibility and reliability. Here, the mechanical properties of low-power flexible complementary metal–oxide–semiconductor (CMOS) logic circuits including inverter, NAND, and NOR are investigated. To fabricate CMOS circuits on flexible polyimide substrates, carbon nanotube (CNT) network films are used for p-type transistors, whereas amorphous InGaZnO films are used for the n-type transistors. The power consumption and voltage gain of CMOS inverters are <500 pW/mm at *V*_*in*_ = 0 V (<7.5 nW/mm at *V*_*in*_ = 5 V) and >45, respectively. Importantly, bending of the substrate is not found to cause significant changes in the device characteristics. This is also observed to be the case for more complex flexible NAND and NOR logic circuits for bending states with a curvature radius of 2.6 mm. The mechanical stability of these CMOS logic circuits makes them ideal candidates for use in flexible integrated devices.

The development of flexible electronic components such as transistors and integrated circuits is essential for the realization of flexible, stretchable, and wearable electronic devices that can be attached to a wide range of surfaces. Many different approaches for the fabrication of high-performance, flexible transistors have been reported previously using organic and/or inorganic materials^1^,^2^,^3^,^4^,^5^,^6^,^7^,^8^,^9^. Due to the processing challenges inherent in the fabrication of thin-film transistors (TFTs) on flexible substrates, most of these reports have focused on the fabrication of these devices, predominantly in deposition^10^,^11^, solution-based processing^12^,^13^,^14^,^15^, and printing methods^3^,^4^,^16^. For reports that do address the mechanical reliability of these devices, these have generally studied the characteristics of single flexible TFT devices (either n-type or p-type)^3^,^6^,^9^,^11^,^14^,^16^,^17^,^18^,^19^,^20^. However, in order to work towards the realization of low-power electrical devices, it is more appropriate to consider the performance of flexible circuit systems such as those based on complementary metal–oxide–semiconductor (CMOS) structures that integrate both n- and p-type TFTs, as opposed to simply measuring the performance of single devices. Several reports have been published that deal with the development of flexible CMOS circuitry, employing fabrication techniques including the film transfer method^1^ or combinations of printing and deposition methods^2^,^21^,^22^,^23^,^24^: these reports present promising results for low-power logic circuits. However, the mechanical flexibility of integrated CMOS logic circuits have yet to be investigated in detail, although testing of standalone TFT devices has been widely reported^1^,^6^,^7^,^17^,^18^. In this study, we report the mechanical flexibility of CMOS logic circuits such as inverter, NAND, and NOR circuits to confirm that they exhibit near-identical electrical characteristics under both flat and bending test conditions. To demonstrate such behavior, flexible polyimide-based TFT devices using amorphous InGaZnO for n-type devices^10^,^24^,^25^ and 99% semiconductor-enriched carbon nanotube (CNT) network films for p-type devices^12^,^24^,^26^ were fabricated and incorporated into the aforementioned logic circuits. This study particularly focuses on the effects of bending on voltage gain and threshold voltage for the CMOS inverters, and the investigation of logic operations for CMOS NAND and NOR circuits.

## Device Fabrication

**Figure 1:**
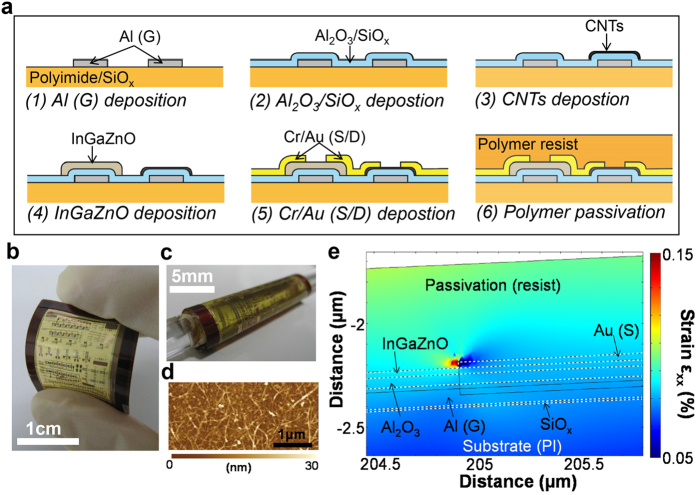
Process and strain distribution. (**a**) Fabrication process of flexible InGaZnO–CNT CMOS logic circuits. Photographs of flexible CMOS circuits under (**b**) bending by hand and (**c**) rolling over a glass bar (~2.6 mm radius). (**d**) AFM image of CNT network film for p-type TFT. (**e**) FEM simulation plot modeling the strain distribution in the InGaZnO channel region under bending (*r* = 2.6 mm).

Figure 1(a) depicts the fabrication process of the flexible InGaZnO–CNT CMOS logic circuits. To form the substrates, a polyimide (PI) solution (Sigma-Aldrich, USA) was spun on a handle wafer of Si/SiO_2_ and cured at 350 °C for 1 hour to form ~10 μm thick PI film. In order to improve the adhesion between the PI film and CMOS device components, a 10 nm thick SiO_*x*_ layer was deposited on the PI surface by electron beam (EB) evaporation. For the gate electrodes of both InGaZnO and CNT TFTs, an aluminum (Al) layer (100 nm thick) was deposited using a sputter tool (ULVAC, Japan), and subsequently patterned using a wet Al etchant. For the gate dielectric, a 50 nm thick layer of Al_2_O_3_ was deposited by atomic layer deposition (ALD) (Arradiance, USA) at 200 °C using the precursors of trimethylaluminium (TMA) and H_2_O, followed by a SiO_*x*_ (10 nm thick) film formed by EB evaporation. We have observed that the presence of the SiO_*x*_ layer on top of Al_2_O_3_ serves to enhance the CNT network deposition, which allows for the realization of devices with high on-currents and mobilities. To further increase the density of the CNT networks^12^, the surface of the SiO_*x*_ layer was cleaned using oxygen plasma and subsequently chemically treated with poly-L-lysine (Sigma-Aldrich) for 5 min. A commercially available 99% semiconductor-enriched CNT solution (Meijo Nano Carbon, Japan) was deposited for 20 min by soaking in the solution and rinsed with deionized (DI) water. CNT deposition and DI water rinse cycles were repeated up to 4 times to form relatively high-density CNT networks. The CNT network films were patterned by oxygen plasma (100 W) for 90 sec. Next, for the n-type channels, 30 nm thick amorphous InGaZnO films were deposited using a sputter tool under a chamber pressure of 13 Pa: this was achieved by controlling argon gas with oxygen gas of 6.4 sccm flow. InGaZnO films were patterned by a lift-off process. Via holes were etched in the Al_2_O_3_/SiO_x_ layers using a buffered hydrofluoric acid (BHF) solution, with subsequent deposition of source (S) and drain (D) electrodes (Cr/Au) carried out by EB evaporation and patterned using a lift-off process. Subsequently, a ~600 nm thick polymer resist (TSMR-V50EL; Tokyo Ohka Kogyo, Japan) layer was spun to passivate the InGaZnO–CNT CMOS circuit, in effect suppressing the strain effect under bending and preventing physical damage. Finally, the devices were detached from the handle wafer. Fig. 1(b,c) show photographs of the flexible CMOS logic circuit devices that were shown not to crack or delaminate under bending. Based on the surface morphology of atomic force microscopy (AFM) image in Fig. 1d, CNT network density is relatively low. By increasing the surface treatment and/or deposition time of CNTs, the density can be increased as the ones reported previously^12^, resulting in that the field effect mobility may be increased more.

## Results and Discussion

To understand the strain distribution under bending, a finite element method (FEM) simulation was conducted. The simulation was only carried out for the InGaZnO film as the mechanical properties of the CNT network film were not known. We speculate that the strain distribution values of the CNT network films under bending are similar to those of InGaZnO; however, further studies into the properties of CNT network films are required to verify this assumption. As shown in Fig. 1(e), under bending with a 2.5 mm curvature radius, the maximum strain in InGaZnO film is ~0.08% tensile strain: such a value may be small enough for the circuit to operate with no changes to its electrical properties. Further reductions to the strain in the InGaZnO film may be attained by using a thicker passivation layer: as observed from Fig. 1(e), this would move the semiconducting layer towards the neutral strain region in the film stack.

**Figure 2:**
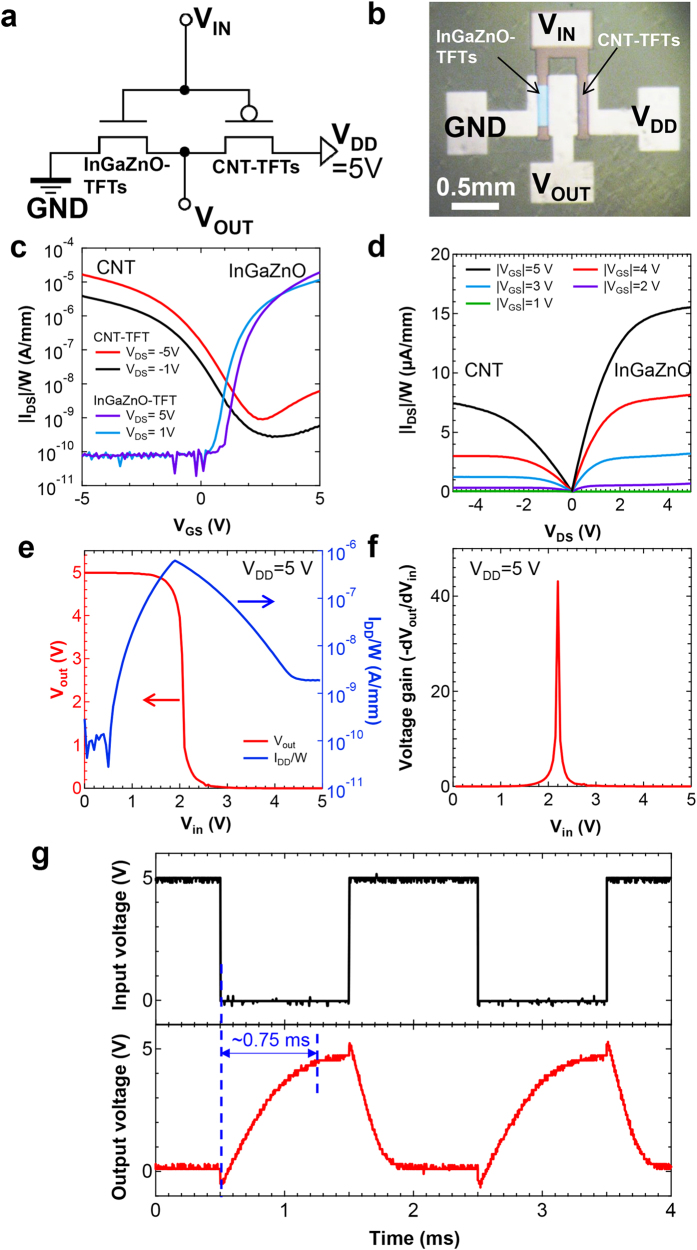
InGaZnO-CNT CMOS inverter. (a) Circuit diagram and (**b**) optical microscope image of a flexible InGaZnO–CNT CMOS inverter. (**c**) *I*_*DS*_ − *V*_*GS*_ and (**d**) *I*_*DS*_ − *V*_*DS*_ curves for n-type InGaZnO TFT and p-type CNT TFT devices. CMOS inverter characteristics: (**e**) output voltage, *V*_*out*_, and drive current normalized with channel width (*W*), *I*_*DD*_/*W*, and (**f**) voltage gain as a function of *V*_*in*_ at *V*_*DD*_ = 5 V. (**g**) Response time of a flexible CMOS inverter.

The electrical properties of the InGaZnO–CNT CMOS inverter were measured prior to testing under bending conditions. The circuit diagram and a photograph of the fabricated CMOS inverter are shown in Fig. 2(a,b). The channel length and width of both InGaZnO and CNT TFTs are 100 μm and 400 μm, respectively, and each current value was normalized according to the channel width. Figure 2(c,d) present the switching and output characteristics of both InGaZnO and CNT TFTs. InGaZnO (CNT) TFTs exhibit a relatively good *I*_*on*_/*I*_*off*_ ratio of >10^5^ (>10^4^) at *V*_*DS*_ = 5 V and a peak field effect mobility of ~5.5 cm^2^/Vs (~2.2 cm^2^/Vs) at *V*_*DS*_ = 1 V. The mobility was extracted from the parallel plate gate model using a measured gate capacitance (*i.e.* Al_2_O_3_/SiO_*x*_) value of ~8.0 × 10^−4^ F/m^2^. Next, the CMOS inverter was characterized as shown in Fig. 2(e,f): the device exhibited a high voltage gain of ~45 at a driving voltage, *V*_*DD*_, of 5 V. Subsequently, the power consumption was calculated using the relation *I*_DD_/*W* × *V*_DD_ ( = 5 V) based on the results of Fig. 2(e): importantly, the power consumption at steady state was found to be <7.5 nW/mm at *V*_*in*_ = 5 V and <500 pW/mm at *V*_*in*_ = 0 V owing to the off-current of two TFT components, ~10^−9^ A/mm for the CNT and ~10^−10^ A/mm for the InGaZnO TFT, values that are comparable to those previously reported for low-power consumption circuits^27^. As the operating speed, Fig. 2g indicates that the rise time of a CMOS inverter is ~0.75 ms by observing the change of output voltage from 0 V to 5 V. The rise speed depends on the FET geometry, and it can be faster by fabricating smaller device if needed.

**Figure 3:**
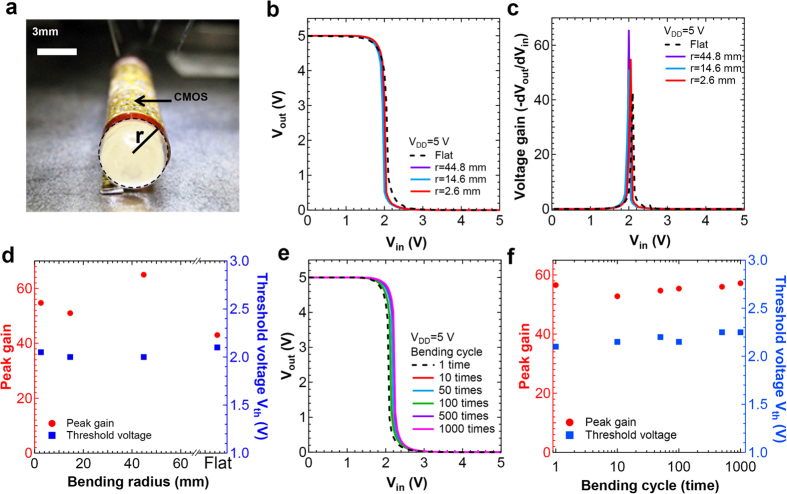
Mechanical properties of CMOS inverter. (**a**) Photograph of the measurement of flexible CMOS circuits and definition of bending radius, *r*. CMOS inverter characteristics of (**b**) *V*_*out*_ − *V*_*in*_ and (**c**) voltage gain at flat and bending conditions (up to *r* = 2.6 mm). (**d**) Compiled peak gain and threshold voltage of the CMOS inverter as a function of bending radius extracted from (**b**,**c**). (**e)** *V*_*out*_ − *V*_*in*_ properties and (f) compiled results of peak gain and threshold voltage for bending cycle tests at *r* < 6 mm for up to 1000 cycles.

Mechanical flexibility of the CMOS inverters is characterized by measuring electrical properties as a function of bending radius, *r*, of the substrate (Fig. 3(a)). First, *V*_*out*_ − *V*_*in*_ at *V*_*DD*_ = 5 V was measured as a function of *r*, up to 2.6 mm (Fig. 3(b,c)). Although there are small differences for electrical properties between each measurement, the peak gain and threshold voltage are relatively constant as shown in the compiled results in Fig. 3(d). This shows that the flexible CMOS inverter is not affected by the strain arising from substrate bending since there is no trend for changes in the electrical properties. This is in good agreement with the FEM simulation of strain distribution as discussed in Fig. 1(e), where the strain in the TFT channel was observed to be small (~0.08%). Since the strain does not significantly affect the performance of the flexible CMOS circuits, small fluctuations for voltage gain and threshold voltage might attribute to a hysteresis of CNT TFTs (Fig. S1). To prevent hysteresis in the characteristics of CNT TFTs, a more effective passivation layer is required, particularly one that can inhibit the diffusion of water molecules into the device^28^. To shed light further on the mechanical reliability of the flexible InGaZnO–CNT CMOS circuit, the device characteristics were measured whilst the circuit was subjected to up to 1000 bending cycles with a bending radius <6 mm: these results are shown in Fig. 3(e,f). Again, although small fluctuations in electrical properties were observed, all measured characteristics including voltage gain and threshold voltage were measured to be relatively stable. These results convey that the fabricated InGaZnO–CNT CMOS inverters are mechanically flexible and reliable given the lack of substantial changes in functional properties on bending.

**Figure 4:**
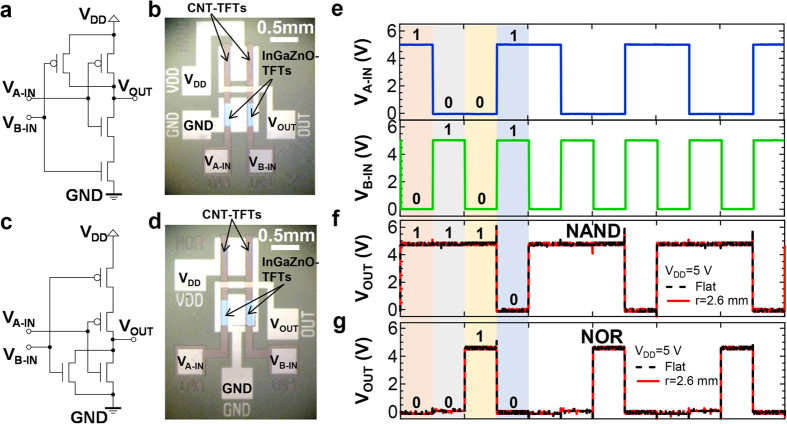
NAND and NOR logic circuits. (**a**) Circuit diagram and (**b**) microscope image of the flexible NAND circuit. (**c**) Circuit diagram and (**d**) microscope image of the flexible NOR circuit. (**e**) Input signal (*V*_*A-IN*_ and *V*_*B-IN*_) for NAND and NOR logic circuits. Represented output voltage of (**f**) NAND and (**g**) NOR circuits at the flat and bending (*r* = 2.6 mm) states when the digital input signals shown in (**e**) are applied.

Considering that more complex circuitry than the CMOS inverter (NOT circuit) is required in many practical applications, CMOS NAND and NOR circuits were also fabricated and characterized under the same bending conditions as used for the CMOS inverter (*r* = 2.6 mm). These two logic circuits are described in Fig. 4(a–d) and used the same channel dimensions (length = 100 μm, width = 400 μm) as the CMOS inverter discussed above. Two voltage input signals (0 V and 5 V) of *V*_A-IN_ and *V*_B-IN_ were used with different frequencies of square pulses with an amplitude of 5 V to create every digital input signal (*i.e.* 00, 01, 10, 11, shown in Fig. 4(e)): for this digital logic circuit, input voltages of 5 V and 0 V are “1” and “0” signals, respectively. Firstly, the flexible CMOS NAND and NOR circuits were confirmed to operate correctly as shown in Fig. 4(f,g). Furthermore, this also shows logic circuit operation under *r* = 2.6 mm bending conditions, showing that the flexible CMOS NAND and NOR circuits, as was the case for the CMOS inverter, possess the same functionality under both flat and bending conditions. From these results, it can be concluded that complex logic circuits can also possess mechanical flexibility with at least hundreds of input operations confirmed by the experiment based on Fig. 4(e–g).

In this study, we fabricated the TFTs with relatively large channel length (100 μm) and width (400 μm). TFTs should be designed for the application depending on how high operating on-current and speed are required. In this platform, if higher parameters are required, several micro meter size for channel length can be used without affecting another performance such as I_on_/I_off_ based on previous studies^12^,^17^. However, smaller channel length may be challenge due to the purity of semiconductor CNTs in the solution (99% semiconductor and 1% metallic CNTs) that creates current paths through the metallic CNTs between S/D electrodes. By developing high purity CNT solution, it may be possible to scale down further in the future.

In summary, we fabricated and characterized flexible CMOS logic circuits by comparing the properties at flat and bending states (up to *r* = 2.6 mm). The results indicate that flexible InGaZnO–CNT CMOS inverter circuits are mechanically stable and function reliably, even after being subjected to multiple bending cycles. Furthermore, it was also confirmed that the more complex CMOS NAND and NOR circuits are mechanically flexible, with no device malfunction observed under bending conditions. Although characterization of standalone TFTs under bending conditions has been widely reported, this is the first demonstration of the operation of flexible CMOS NAND and NOR circuits under such conditions, confirming that such components are insensitive to significant degrees of external stress. We believe that this demonstration of mechanically flexible CMOS logic circuits opens the door towards realizing low-power, high-speed, flexible CMOS systems that are likely to be key components in the next generation of portable electronic devices. Finally, next step for the practical use of flexible circuits should be the developments of both highly integrated circuits with high reliability and low-cost process. Since the flexible devices target the macroscale electronics unlike Si-based electronics, the conventional Si-process infrastructure should not be used in terms of the fabrication cost. To address this issue, printing technique for the flexible CMOS circuits is an important role for the future flexible electronics.

## Additional Information

**How to cite this article**: Honda, W. *et al.* Mechanically Flexible and High-Performance CMOS Logic Circuits. *Sci. Rep.* **5**, 15099; doi: 10.1038/srep15099 (2015).

## Supplementary Material

Supplementary Information

## References

[b1] Ahn J. H. *et al.* Heterogeneous Three-Dimensional Electronics by Use of Printed Semiconductor Nanomaterials. Science 314, 1754–1757 (2006). 10.1126/science.1132394 17170298

[b2] Chen H., Cao Y., Zhang J. & Zhou C. Large-Scale Complementary Macroelectronics using Hybrid Integration of Carbon Nanotubes and IGZO Thin-Film Transistors. Nat Commun. 5, 4097 (2014). 24923382 10.1038/ncomms5097

[b3] Javey A., Nam S., Friedman R. S., Yan H. & Lieber C. M. Layer-by-Layer Assembly of Nanowires for Three-Dimensional, Multifunctional Electronics. Nano Lett. 7, 773–777 (2007). 17266383 10.1021/nl063056l

[b4] Kiriya D. *et al.* Design of Surfactant-Substrate Interactions for Roll-to-Roll Assembly of Carbon Nanotubes for Thin-Film Transistors. J. Am. Chem. Soc. 136, 11188–11194 (2014). 25019509 10.1021/ja506315j

[b5] Takahashi T. *et al.* Parallel Array InAs Nanowire Transistors for Mechanically Bendable, Ultrahigh Frequency Electronics. ACS Nano 10, 5855–5860 (2010). 20845916 10.1021/nn1018329

[b6] Takei K. *et al.* Nanowire active-matrix circuitry for low-voltage macroscale artificial skin. Nat. Mater. 9, 821–826 (2010). 20835235 10.1038/nmat2835

[b7] Wang C. *et al.* Self-Aligned, Extremely High Frequency III-V Metal-Oxide-Semiconductor Field-Effect Transistors on Rigid and Flexible Substrates. Nano Lett. 12, 4140–4145 (2012). 22746202 10.1021/nl301699k

[b8] Moon H. *et al.* Synthesis of Ultrathin Polymer Insulating Layers by Initiated Chemical Vapour Deposition for Low-Power Soft Electronics. Nat. Mater. 14, 628–635 (2015). 25751074 10.1038/nmat4237

[b9] Kaltenbrunner M. *et al.* An ultra-lightweight design for imperceptible plastic electronics. Nature 499, 458–463 (2013). 23887430 10.1038/nature12314

[b10] Nomura K. *et al.* Room-Temperature Fabrication of Transparent Flexible Thin-Film Transistors using Amorphous Oxide Semiconductors. Nature 432, 488–492 (2004). 15565150 10.1038/nature03090

[b11] Petti L. *et al.* Low-Temperature Spray-Deposited Indium Oxide for Flexible Thin-Film Transistors and Integrated Circuits. Appl. Phys. Lett. 106, 092105 (2015).

[b12] Takahashi T., Takei K., Gillies A. G., Fearing R. S. & Javey A. Carbon nanotube active-matrix backplanes for conformal electronics and sensors. Nano Lett. 11, 5408–5413 (2011). 22050705 10.1021/nl203117h

[b13] Choi C.-H. *et al.* Fabrication of High-Performance, Low-Temperature Solution Processed Amorphous Indium Oxide Thin-Film Transistors using a Volatile Nitrate Precursor. J. Mater. Chem. C 3, 854–860 (2015).

[b14] Sun D. M. *et al.* Flexible High-Performance Carbon Nanotube Integrated Circuits. Nat. Nanotech. 6, 156–161 (2011). 10.1038/nnano.2011.1 21297625

[b15] Seo J. S. *et al.* Solution-Processed Flexible Fluorine-Doped Indium Zinc Oxide Thin-Film Transistors Fabricated on Plastic Film at Low Temperature. Sci. Rep. 3, 2085 (2013). 23803977 10.1038/srep02085 PMC3694285

[b16] Lau P. H. *et al.* Fully Printed, High Performance Carbon Nanotube Thin-Film Transistors on Flexible Substrates. Nano Lett. 13, 3864–3869 (2013). 23899052 10.1021/nl401934a

[b17] Wang C. *et al.* Extremely Bendable, High-Pperformance Integrated Circuits Using Semiconducting Carbon Nanotube Networks for Digital, Analog, and Radio-Frequency Applications. Nano Lett. 12, 1527–1533 (2012). 22313389 10.1021/nl2043375

[b18] Yu W. J. *et al.* Small Hysteresis Nanocarbon-Based Integrated Circuits on Flexible and Transparent Plastic Substrate. Nano Lett. 11, 1344–1350 (2011). 21322606 10.1021/nl104488z

[b19] Sun D. M. *et al.* Mouldable All-Carbon Integrated Circuits. Nat. Commun. 4, 2302 (2013). 23917348 10.1038/ncomms3302

[b20] Cao Q. *et al.* Medium-scale carbon nanotube thin-film integrated circuits on flexible plastic substrates. Nature 454, 495–500 (2008). 18650920 10.1038/nature07110

[b21] Hong K. *et al.* Aerosol Jet Printed, Sub-2 V Complementary Circuits Constructed from P- and N-Type Electrolyte Gated Transistors. Adv. Mater. 26, 7032–7037 (2014). 24975133 10.1002/adma.201401330

[b22] Nam S., Jiang X., Xiong Q., Ham D. & Lieber C. M. Vertically Integrated, Three-Dimensional Nanowire Complementary Metal-Oxide-Semiconductor Circuits. Proc. Natl. Acad. Sci. U S A 106, 21035–21038 (2009). 19940239 10.1073/pnas.0911713106 PMC2783010

[b23] Gao P., Zou J., Li H., Zhang K. & Zhang Q. Complementary logic gate arrays based on carbon nanotube network transistors. Small 9, 813–819 (2013). 23208943 10.1002/smll.201201237

[b24] Honda W. *et al.* High-Performance, Mechanically Flexible, and Vertically Integrated 3D Carbon Nanotube and InGaZnO Complementary Circuits with a Temperature Sensor. Adv. Mater. 6, 4674–4680 (2015). 26177598 10.1002/adma.201502116

[b25] Liu P.-T., Chou Y.-T., Teng L.-F. & Fuh C.-S. High-Gain Complementary Inverter with InGaZnO/Pentacene Hybrid Ambipolar Thin Film Transistors. Appl. Phys. Lett. 97, 083505 (2010).

[b26] Wang C. *et al.* Wafer-Scale Fabrication of Separated Carbon Nanotube Thin-Film Transistors for Display Applications. Nano Lett. 9, 4285–4291 (2009). 19902962 10.1021/nl902522f

[b27] Huang T.-C. *et al.* Pseudo-CMOS: A Design Style for Low-Cost and Robust Flexible Electronics. IEEE Trans. Electron Dev. 58, 141–150 (2011).

[b28] Ha T. J., Kiriya D., Chen K. & Javey A. Highly Stable Hysteresis-Free Carbon Nanotube Thin-Film Transistors by Fluorocarbon Polymer Encapsulation. ACS Appl. Mater. Interfaces 6, 8441–8446 (2014). 24796606 10.1021/am5013326

