# Supplementary material for: Mechanically Flexible and High-Performance CMOS Logic Circuits
Source: Sci Rep. 2015 Oct 13;5:15099. doi: 10.1038/srep15099 (PMC4602193; doi:10.1038/srep15099)
Supplement: Supplementary Information [file srep15099-s1.pdf]

# SUPPLEMENTARY INFORMATION

## Mechanically Flexible and High-Performance CMOS Logic Circuits

Wataru Honda, Takayuki Arie, Seiji Akita & Kuniharu Takei*

*Department of Physics and Electronics, Osaka Prefecture University, Sakai, Osaka 599-8531, Japan*

*Corresponding author: takei@pe.osakafu-u.ac.jp

## Hysteresis of CNT and InGaZnO TFTs

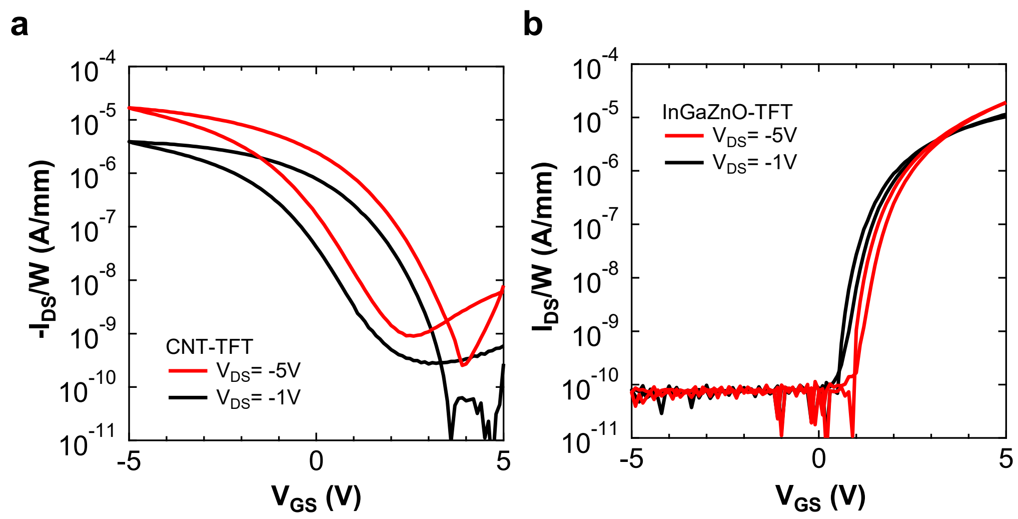


**Figure S1. Hysteresis of $I_{DS}$-$V_{GS}$ curves.** (a) CNT TFT and (b) InGaZnO TFT.
